# Supplementary material for: Saccharomyces boulardii (CNCM I-745) improves intestinal damage in sepsis by remodeling intestinal flora structure
Source: Front Cell Infect Microbiol. 2025 Jul 31;15:1602792. doi: 10.3389/fcimb.2025.1602792 (PMC12350329; doi:10.3389/fcimb.2025.1602792)
Supplement: Supplementary file 1 [file DataSheet1.zip › summary/summary/6_Taxonomy_community/1_Taxonomy_profiling/7_Species/Group/Species_abund_top30_Group.heatmap.pdf]

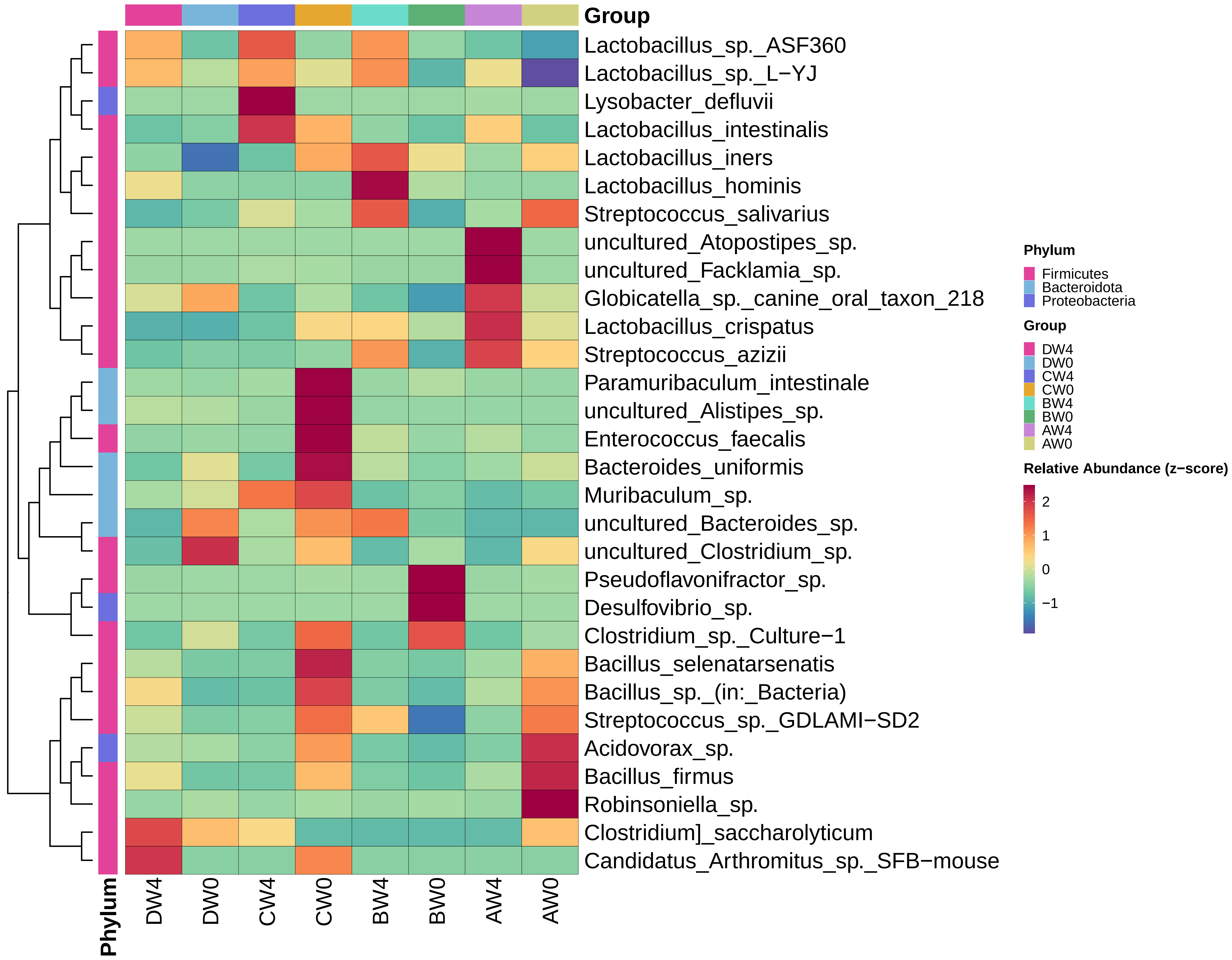
Group
Lactobacillus_sp._ASF360
Lactobacillus_sp._L−YJ
Lysobacter_defluvii
Lactobacillus_intestinalis
Lactobacillus_iners
Lactobacillus_hominis
Streptococcus_salivarius
uncultured_Atopostipes_sp.
uncultured_Facklamia_sp.
Globicatella_sp._canine_oral_taxon_218
Lactobacillus_crispatus
Streptococcus_azizii
Paramuribaculum_intestinale
uncultured_Alistipes_sp.
Enterococcus_faecalis
Bacteroides_uniformis
Muribaculum_sp.
uncultured_Bacteroides_sp.
uncultured_Clostridium_sp.
Pseudoflavonifractor_sp.
Desulfovibrio_sp.
Clostridium_sp._Culture−1
Bacillus_selenatarsenatis
Bacillus_sp._(in:_Bacteria)
Streptococcus_sp._GDLAMI−SD2
Acidovorax_sp.
Bacillus_firmus
Robinsoniella_sp.
Clostridium]_saccharolyticum
Candidatus_Arthromitus_sp._SFB−mouse
Phylum
DW4
DW0
CW4
CW0
BW4
BW0
AW4
AW0
Phylum
Firmicutes
Bacteroidota
Proteobacteria
Group
DW4
DW0
CW4
CW0
BW4
BW0
AW4
AW0
Relative Abundance (z−score)
2
1
0
−1
